# Supplementary material for: Evolution of heterogeneous perceptual limits and indifference in competitive foraging
Source: PLoS Comput Biol. 2021 Feb 23;17(2):e1008734. doi: 10.1371/journal.pcbi.1008734 (PMC7901736; doi:10.1371/journal.pcbi.1008734)
Supplement: Attachment — Submitted filename: 20201012 Mann.pdf [file pcbi.1008734.s003.pdf]

Review for Mann, “Evolution of heterogeneous perceptual limits and indifference in competitive foraging”
PCOMPBIOL-D-20-01455

The author shows how deviations from the Ideal Free Distribution (leading rich resources to be underexploited and poor resources to be overexploited) may result from sensory costs. Costs can generate multiple stable strategies including a subpopulation of uninformed individuals. The author proposes a multilevel model to demonstrate the emergence of coexisting strategies and provides a clear, intuitive explanation for how this phenomenon arises supported by numerical simulation. Overall, the manuscript is written well, and I recommend publication pending a few minor points.

- In the introduction, it seems worthwhile to mention the role of noise in modulating collective decisions or conversely how heterogeneity in individual might be represented using noise. There is a recent preprint in *Nature Physics* about this (Jhawar et al. 2020), but there other papers that the author may consider citing. Furthermore, one might also consider mentioning spatially local forms of social information that can lead to nontrivial global dynamics such as fragmentation. I see the author has cited several of Couzin’s papers—so there is Couzin’s 2005 *Nature* paper showing bifurcations in group direction—but there are also papers on bird blocks (Cavagna et al. or Ballerini et al. in *PNAS* that seem relevant).
- I would suggest using a variable other than δ for the perceptual sensitivity threshold since that is usually used for infinitestimals.
- While the cost function is reasonable, is there a reason for why it does not diverge at perfect precision? One might make an information-theoretic argument that infinite precision leads to diverging cost. My sense is that this will not change your general results, but perhaps some form of this point could be mentioned in lines 116-117.
- Line 137 typo: “strategy, [I] look for”
- Lines 208-209: The justification makes sense. I’m wondering if there is a simple calculation for the correction. Just curious and am not suggesting this is necessary to address for the final draft.
- Is there a particular reason the simulations are instantiated at delta=0? This is introduced without explanation in Line 200. Does the simulation take longer to settle if we started at delta=infty?
- Some additional citations seem warranted for the concluding paragraph such as for the sentence on Lines 331-332.
- I cannot run the attached code because external code is sourced `source(‘~/Dropbox/R/MyRUtils/image.scale.R’)`.
- I am not sure if this is intentional but would the function `evole_population` be named `evolve_population`?

I enjoyed reading this paper!
