# Supplementary material for: Evolution of heterogeneous perceptual limits and indifference in competitive foraging
Source: PLoS Comput Biol. 2021 Feb 23;17(2):e1008734. doi: 10.1371/journal.pcbi.1008734 (PMC7901736; doi:10.1371/journal.pcbi.1008734)
Supplement: Attachment — Submitted filename: PLoSCB_R2R.docx [file pcbi.1008734.s004.docx]

**Thank you for the opportunity to submit a revised version of the attached manuscript, and my thanks to the reviewers for their constructive critique of the previous version. Below I have given a point-by-point reply to each review.**

----------------------------------------------------------------------------------------------------------------------

**Reviewer #1:** The author discusses in his paper the evolution of perceptual limits allowing agents to distinguish patches of different quality. The author introduces a simple but rather generic mathematical model, discusses and analyzes it first analytically in the equilibrium case, and finally performs some agent-based simulations to test the predictions of the theory. A core results is the emergence of evolutionary branching for certain parameter ranges, where a co-existence of agents with no perceptual sensitivity and agents with perceptual capability tuned to the actual differences in resource value can be observed. Overall, this appears to be solid work, with no obvious flaws in the math and reasonable results both of the analytical analysis and the numerical simulations. The manuscript is well written and should be comprehensible to interested readers. I did enjoy reading the paper and it yields some interesting results, as well as provides possible explanations for evolution of heterogeneous perceptual constraints, which could explain empirical deviations from the Ideal Free Distribution. In summary the manuscript, appears potentially suited for publication with PLoS Comp. Biol. However, I have a number of (minor) revision suggestion for the author:

- There are some open questions regarding the generality. The overall model assumes a simple linear dependency on the value difference, however it may be that there is e.g. a saturation of fitness benefits with increasing value (e.g. described by a logistic function). Would this change the qualitative picture?

**The core qualitative results should remain unchanged under most transformations between reward and fitness. If fitness = g(R) – c, where g() is some modulating function such as the logistic suggested by the reviewer, then the steady state condition for the replicator equation specifies that g(R) – c must be the same for all occupied states. Following the original argument in the ms, there must be *some* difference in reward between the patches at equilibrium in the lower game, \Delta R. For any two values of the sensitivity threshold tau (changed in response to reviewer) below \Delta R, the higher value will have greater fitness as c(tau) is monotonic and g(R) will be the same for both.Similarly for any two values of tau greater than \Delta R the higher will have greater fitness – this maintains the bifurcation into informed and uniformed agents. Since g(R) – c must be the same for the informed and uniformed agents in the steady state of the upper game, the informed agents will converge to a numerically different value of tau, depending on the choice of g(). The analogous arguments hold in the same way for the case of multiple patch ratios**

- The model assumes strictly monotonously decreasing cost functions with delta, based on the mathematical expression in line 112. Below the definition of the cost function (Eq. 4) the author says that the results are general and other cost functions can be substituted. Further below, following Eq. 8, the authors it bases that results from c decreasing monotonically with delta. It appears intuitively reasonable that the results should be general for strictly monotonously decreasing cost function. So convexity/concavity of the function does not play a role. This should be clearly stated at the latest following Eq. 4.

**After eqn 4 I have added the following text:**

***The cost function I have chosen is also convex, but the analytical results I will present do not rely on this property.***

Here, however one can easily imagine that the cost function is not strictly monotonously decreasing but only monotonously decreasing: c(delta')<=c(delta) <-> delta'>delta, for example if at some point the difference is so large that distinguishing the patches does not incur any additional costs above a critical delta_crit. If this may change the results qualitatively, this should be clearly stated. The naive expectation would be simply the non-responsive simply become responsive with delta_crit. Thus as long as all deltas are below delta_crit then the results are the same (mathematically this last point is closely related to my first comment)

**If one imposes a non-decreasing cost function for a threshold above that of the informed agents, the only change that occurs is that the uniformed agents no longer drift systematically towards a threshold of infinity. Instead, once they reach the plateau of the cost function, further loss of sensitivity occurs only by diffusion rather than by selection. This leads to no qualitative difference in outcome, since these agents all still remain above the equilibrium difference in rewards between the patches.**

**If the plateau occurs for a threshold below the equilibrium reward difference then all agents evolve the same finite level of perception at this plateau.**

**I have added an additional paragraph in which I note that I rely on the strict monotonicity of the cost function and showing how the mathematical argument can be adapted to the case where this is violated. I also point to a new supplementary figure (Figure S1) that illustrates the results when the cost function plateaus beyond a certain sensitivity using the agent-based model.**

- The author refers to the non-responsive agents in the abstract, introduction, and discussion, as "free-riders". Classically free riding is defined by agents benefiting from some public good but not paying for it. Here the public good are the resources but the agents don't pay for access to resources but only for the ability to distinguish their quality. They just don't care about the value differences. In general, free-riding is also associated with the existence of social dilemmas, where the global (or group) optima differ from evolutionary stable strategies, with the letter leading to suboptimal reward at the group level. It is unclear on first glance whether this is the case here. Note, the scenario considered here is fundamentally different from the collective migration work by Guttal and Couzin cited in the manuscript. There, a fraction of agents evolves to not to detect the environmental gradient but only to be social, thus they free-ride on few informed agents that evolved gradient detection, with directional information being the public good generated by the informed agents. Thus, I am not sure this term is really justified here. Either the author should explain better why this is a form of free-riding or rewrite the corresponding parts.

**I agree that the phenomenon here is not the classic form of ‘free riding’ as described by the reviewer. One can argue that the uninformed agents free ride on the provision of a social good: the creation of an environment that provides a good foraging return on either patch. However, as the reviewer suggests, there is no social dilemma here; the informed agents do not lose out. Rather than argue the analogy at the potential cost of confusion, I have removed references to free riding throughout**

- In the motivation there is strong emphasis on empirically observed under matching of IFD, which is largely based on the review of Kennedy and Gray, 1993. The author should have a look at: Milinski, M. (1994). Ideal free theory predicts more than only input matching: a critique of Kennedy and Gray's review. Oikos, 163-166. The author correctly states that perceptual constraints have been suggested as an explanation for the under matching of the IFD. However, given this strong emphasis on this "flaw" of IFD, I feel the author should acknowledge the many other mechanisms that could be responsible for it (see e.g. references in Milinsky, 1994). Maybe even discuss briefly how the mechanisms discussed in this manuscript could be distinguished from these other mechanisms experimentally, i.e. how his theory could be tested.

**Thank you for the pointer to Milinski’s rebuttal to Kennedy and Gray. I have noted that Milinski correctly asserts that there are in fact many IFDs depending on how agents interfere with each other’s foraging. As Milinski states, this means that under matching is not a refutation of the IFD *per se,* only a particular (and common) form of IFD, which I have endeavoured to make clear in both the introduction and discussion. I have also made more explicit reference to research on other mechanisms such a movement costs, and clarified the purpose of this study, which is not to uniquely explain under matching, but to investigate the evolution of perceptual constraints (which surely do exist) in competitive foraging, motivated by their inclusion in previous models (where they have been invoked as one mechanism for undermatching).**

- In the discussion the author refers to some previous work without providing the reference at this point, as e.g. line 278-281. These should be provided.

**I have added the appropriate references here**

----------------------------------------------------------------------------------------------------------------------

**Reviewer #2:** The manuscript by Mann et al is an interesting study on how evolution favours heterogeneous perceptual strategies in a foraging context. The article writing is lucid. It has analytical calculations whose conclusions are supported by agent-based simulations.

The author has done a really nice job of putting the study and its results in the context of existing body of work. The results by Mann in this manuscript are broadly consistent with the frameworks on producer scrounger dynamics as well as differential navigation strategies — as clearly explained by Mann. The author claims new and broadly applicable insights — especially on novel implications on ideal free distribution theory. Broadly speaking, if one requires a trait to utilise a resource and if that trait has some cost, for a wide-range of conditions, we find an evolution of differential strategies. In that sense, the results claimed by the author fall within the known framework of the evolution of differential strategies. Therefore, the novelty or newness of the results shown here are not clear to me.

**It is well-understood that requiring a trait to utilise a resource will lead to differential strategies. The novelty in this work is of three forms: (i) applying this logic specifically to the case of perceptual abilities within a IFD-type foraging game, to explain the origin and form of perceptual limits previously imposed exogenously. This analysis reveals that the assumption of identical limits previously used in these models is likely unrealistic; (ii) showing the emergence of a particular ‘indifferent’ foraging strategy – that is, not simply a range of perceptual abilities but the emergence of a class of agent that loses all perception (this is party analogous to the cited example from navigation, but as far as I am aware is novel in the context of foraging models; and (iii) to reveal the existence of multiple distinct ‘niches’ along a single trait dimension (perceptual accuracy) emerging from the environmental variability, rather than from the availability of multiple traits. In particular, point (ii) is relevant to competitive contexts well beyond the case study of animal foraging. As indicated in the discussion, many games result in equilibria where rewards are equal across strategies; these are all susceptible to invasion by agents who do not pay the cognitive or perceptual cost of identifying an optimal strategy.**

**In the light of comments by reviewer 1, I have removed description of the phenomenon here as a ‘producer-scrounger dynamic’, since I agree that while there is an analogy between the two, this does not strictly match what is going on here; the ‘informed’ agents do not produce a social good for the ‘uninformed’ agents to scrounge.**

What I found interesting was the exploration of how the strategies evolve in variable environmental context. Compared to the existing works on the evolution of heterogeneous strategies in a foraging context, I think the main new biological finding seems to be in this section on Variable environments. However, this section is not explored as much in terms of generality. Mann has modelled this variable environment by assuming that resource parameter r changes between two discrete values. Previous studies had only shown bimodal heterogenous strategies whereas this study shows that even trimodal strategies are possible; I think the author claims that it can be more than three if the variability of r happens between three values or more, but do not provide any analytical or numerical treatment for the same. Therefore, my questions are: Is this result general? What if there was continuous stochastic variation in resource variability parameter r? What if the number of discrete jumps between resources were more than two?

**I agree this is an interesting result and worthy of further exploration. While the analytical results show the existence of an increasing number of sensitivity thresholds at equilibrium as the number of patch ratios is increased, I had not shown how this plays out at the level of the for a specific distribution of patch rations or in the agent -based model. Based on the reviewer’s suggestion I have constructed an experiment which approximates a continuous distribution of patch ratios (in fact 100 distinct ratios). To choose these, I have argued for a reasonable probability distribution over patch ratios such that r/(1+r) is uniformly distributed between 0.5 and 1 (one could equally we choose between 0 and 1, but I retain the convention that r is the ratio of the larger patch to the smaller). These results, including a new figure (fig 6) are now presented in the manuscript, and show in both the analytical and agent-based models the emergence of a broad range of sensitivity thresholds (for appropriate parameter values) alongside the indifferent agents.**

In summary: I think that the evolution of differential strategies in a foraging context are sort of known. It could be a much stronger if the focus is on the evolution of resource utilisation/extraction/finding strategies in the context of variable environments.

Minor point: Here is another such paper showing the evolution of differential strategies in a foraging/resource extraction context: Joshi, Jaideep, Åke Brännström, and Ulf Dieckmann. "Emergence of social inequality in the spatial harvesting of renewable public goods." PLoS computational biology 16.1 (2020): e1007483.

**Thank you for this reference, I have now included this in my discussion.**

----------------------------------------------------------------------------------------------------------------------

**Reviewer #3:** The author shows how deviations from the Ideal Free Distribution (leading rich resources to be underexploited and poor resources to be overexploited) may result from sensory costs. Costs can generate multiple stable strategies including a subpopulation of uninformed individuals. The author proposes a multilevel model to demonstrate the emergence of coexisting strategies and provides a clear, intuitive explanation for how this phenomenon arises supported by numerical simulation. Overall, the manuscript is written well, and I recommend publication pending a few minor points.

•In the introduction, it seems worthwhile to mention the role of noise in modulating collective decisions or conversely how heterogeneity in individual might be represented using noise. There is a recent preprint in *Nature Physics* about this (Jhawar et al. 2020), but there other papers that the author may consider citing. Furthermore, one might also consider mentioning spatially local forms of social information that can lead to nontrivial global dynamics such as fragmentation. I see the author has cited several of Couzin’s papers—so there is Couzin’s 2005 *Nature* paper showing bifurcations in group direction—but there are also papers on bird blocks (Cavagna et al. or Ballerini et al. in *PNAS* that seem relevant).

**I have added to the first paragraph of the introduction to note the role of noise in collective behaviour, with reference to Jhawar et al 2020 and other closely related papers exploring the same phenomenon:**

***Both theoretical and empirical evidence has highlighted the importance of `noise' in collective behaviour. This noise may result from unobserved differences between individuals that cause them to act in unpredictable ways in apparently identical situations***

**I have also added a short note in a later paragraph to note that:**

***Contingent differences in information may be one source of variation (e.g. the position of an agent in its environment may lead to specific knowledge that others lack)***

• I would suggest using a variable other than for the perceptual sensitivity threshold since that is usually used for infinitestimals.

**I agree – I have changed the sensitivity to be denoted as tau**

• While the cost function is reasonable, is there a reason for why it does not diverge at perfect precision? One might make an information-theoretic argument that infinite precision leads to diverging cost. My sense is that this will not change your general results, but perhaps some form of this point could be mentioned in lines 116-117.

This would indeed be equally reasonable. The main reason I have chosen not to do this is to avoid having the model make infinite precision impermissible. That is, I did not want to make perceptual limits a trivial consequence of the cost function. After eqn 4 I have added the following text

*In particular, principle (iii) above could justifiably be removed, such that perfect perception incurs an infinite cost and is therefore not possible. I retain this condition to avoid making perceptual limits tautological.*

• Line 137 typo: “strategy, [I] look for”

**Fixed**

• Lines 208-209: The justification makes sense. I’m wondering if there is a simple calculation for the correction. Just curious and am not suggesting this is necessary to address for the final draft.

**As a crude estimate, it seems reasonable that this correction would be on the order 1-3 times the mutation parameter (the standard deviation of changes in delta from one generation to the next). However, it isn’t clear from looking at a few examples that there is a consistent multiple that fits particularly well for a variety of mutation parameters.**

• Is there a particular reason the simulations are instantiated at delta=0? This is introduced without explanation in Line 200. Does the simulation take longer to settle if we started at delta=infty?

**Thank you for raising this issue. I had started simulations with all agents at 0 simply because this seemed like a natural starting point, but of course all agents having no perception is equally plausible. Indeed, the dynamics to equilibrium are quite different, as I now address in Figure 2 and the corresponding text. Essentially, from delta =0, the agents can fall down the cost gradient to the first equilibrium level, then a small mutation over this point allows them to follow the natural gradient to infinity. In reverse the process is resisted by the same gradient, such that with the same mutation level the agents remain at infinity. Increasing the mutation rate allows the agents to mutate to the lower equilibrium level of delta against the selection gradient, whereupon they can stably remain at this equilibrium. As noted in the discussion, this has interesting implications for what we can expect to observe in instances where perceptive abilities have been gained or lost**

• Some additional citations seem warranted for the concluding paragraph such as for the sentence on Lines 331-332.

**I have added appropriate references for the traffic and queueing examples**

• I cannot run the attached code because external code is sourced `source(‘~/Dropbox/R/ MyRUtils/image.scale.R’)`.

**Fixed**

• I am not sure if this is intentional but would the function `evole_population` be named `evolve_population`?

**Fixed**

I enjoyed reading this paper!
