# Supplementary material for: Evolution of heterogeneous perceptual limits and indifference in competitive foraging
Source: PLoS Comput Biol. 2021 Feb 23;17(2):e1008734. doi: 10.1371/journal.pcbi.1008734 (PMC7901736; doi:10.1371/journal.pcbi.1008734)
Supplement: Attachment — Submitted filename: PLoSCB_R2R2.docx [file pcbi.1008734.s005.docx]

Thank you for the opportunity to submit this revised copy of the attached manuscript, with minor corrections in line with the reviewer comments as laid out below:

**Reviewer #1:**

Thank you for pointing out the omission of the random switching term for the case where tau > \Delta R.

I have corrected this omission and rewritten the equation in the form of multiple cases to make it easier to read. Following the reviewer suggestion, I have used the Heaviside function rather than indicator functions. I think the reviewer mistook the rate 1 in the text for rate I, whereas the I’s in the equation were supposed to be indicator functions (which I had not specified). I have thus revised the text to spell out ‘one’ and have removed the indicator functions in favour of the Heaviside function, which should clarify this.

**Reviewer #3:**

I have corrected the typo in the caption of Figure S1.
